# Understanding the function of social capital among Mexican and Chinese immigrants in Southern California: A qualitative study

**DOI:** 10.1016/j.ssmqr.2023.100247

**Published:** 2023-03-04

**Authors:** Altaf Saadi, Brenda Morales, Lei Chen, May Sudhinaraset

**Affiliations:** a Department of Neurology, Massachusetts General Hospital, Harvard Medical School, Boston, MA, USA; b Department of Social Welfare, Luskin School of Public Affairs, University of California Los Angeles, Los Angeles, CA, USA; c Department of Community Health Sciences, Fielding School of Public Health, University of California Los Angeles, Los Angeles, CA, USA

**Keywords:** Social capital, Social networks, Immigrant, Immigrant health, Disparities

## 1. Introduction

Social capital is crucial to health and wellbeing (Berkman et al., 2000; Kawachi & Berkman, 2001; Thoits, 2011). Social capital, defined as the resources that are derived from social networks and social relationships, can enable exchange of health information, spread knowledge, establish cultural norms, health beliefs and normative behaviors, and facilitate emotional or financial support (Berkman, 1986). These benefits can occur at the individual or institutional level, although negative (i.e. harmful) associations with health outcomes have also been documented (Villalonga-Olives & Kawachi, 2017). Links to institutions are a type of linking capital, giving individuals and communities access to networks or groups with greater access to power or status. while interpersonal links can be either bonding (networks among friends, family, neighbors who are similar in the way they define themselves) or bridging (between those from different demographic and spatial groups) (Szreter & Woolcock, 2004). In other words, linking social capital is related to the vertical relations of authority whereas bridging and bonding social capital are derived from horizontal relations.

Latinx and Asian immigrants represent the largest and fasting growing immigrant populations in the United States (US), respectively (Budiman & Ruiz, 2021). They face significant barriers to healthcare, with factors such as lower socioeconomic background, limited English proficiency, immigration status, and stigma and marginalization influencing their vulnerability in the healthcare sector (Derose et al., 2007). Social capital, or lack thereof as immigrants’ social connections change with migration, has been identified as another dimension of immigrants’ vulnerability (Derose et al., 2007). In fact, lack of bonding, bridging, and linking ties have been associated with delays in receipt of medical care, decreased help-seeking behavior, decreased health promotion activities like physical exercise, and decreased self-management of chronic diseases among Latinx and Chinese immigrants (Clough et al., 2013; Nieminen et al., 2013; Schenk et al., 2018; Waverijn et al., 2017); conversely, the presence of these connections has been associated with improved health literacy, easier navigation of the health system including for immigrants’ U.S.-born children, better health outcomes, and greater trust in societal sectors beyond health, like education and employment or health.(Fry-Bowers et al., 2014; Jang et al., 2015; Madden, 2015; Mohseni & Lindstrom, 2007; Valencia-Garcia et al., 2012). Relevant mechanisms include facilitating the acquisition of useful health-related information, the gaining of instrumental support to meet tangible needs like transportation or meal preparation, and social reinforcement of health behaviors. The stress-buffering effects of social capital may be particularly salient for immigrant populations due to the unique stressors they face i.e., social disadvantage, lack of employment, lack of access to and equities in health, and the weakening of ties with people in their home country necessitating the need to establish and strengthen social networks in their new communities(Massey, Goldring, and Durand 1994; Morey et al., 2021). In other words, social capital can be important to overcoming barriers and increasing access to healthcare.

Yet while Latinx and Asian immigrants experience distinct racialization processes and migration experiences, few studies have empirically examined how social capital may differ across these two groups. For example, Asian immigrants as a group tend to demonstrate higher educational attainment, income and better English proficiency when compared to other immigrant populations such as Latinx immigrants(Krogstad & Radford, 2018); this may facilitate access to, and navigation of, social capital at multiple levels. Further, there are differences within Latinx and Asian immigrant communities themselves, given incredible within-group heterogeneity, that are lost when health research treats Latinx and Asian immigrants as homogenous.

Studies assessing how social capital is derived and navigated have also traditionally focused on healthcare, rather than taking a cross-sectoral approach, despite the reality that immigrants’ lives are influenced by policies across multiple sectors that collectively impact immigrant health and health service use(Wallace et al., 2018).

The current study aims to fill these gaps by examining 1) how linking, bridging, and bonding social capital facilitate access within and across four policy sectors; and 2) what factors buffer or exacerbate barriers experienced in accessing resources across these policy sectors in the context of unevenly distributed social capital. We examine four sectors through which state-level immigrant policies have been shown to impact health: (1) Health and social welfare; (2) Education; (3) Labor & employment; (4) Legal, including legal services as well as law and immigration enforcement(Wallace et al., 2018). We focus on Mexican, Chinese, and Taiwanese immigrants as specific subgroups within the Latinx and Asian immigrant communities to account for heterogenous experiences living in the U.S. within these broad categories, including potential cultural differences that may influence access to, and engagement with, social capital processes.

## 2. Methods

### 2.1. Study design and sample

The Research on ImmiGrant HealTh and State policy (RIGHTS) study was designed to understand the experiences related to immigrant policies by Latinx and Asian immigrants who live in California and how those experiences impact their health and access to health care. Both groups have a long history of migration to the United States, and to California in particular, and have historically experienced social, political, and program exclusions created by federal and state-level immigrant policies(-Perreira & Pedroza, 2019). The vast majority of the 10.5 million immigrant adults living in California were born in Latin America (49%) or Asia (38%)(Ruggles et al., 2020).

The qualitative component of the RIGHTS study recruited a convenience sample of Mexican and Chinese and Taiwanese immigrant adults from Los Angeles and Orange counties. Although California is a state that has become increasingly friendly to immigrants, these two counties represent different approaches to immigrants with Los Angeles County (LAC) generally being more immigrant-friendly than Orange County (OC)(Pastor et al., 2012). Los Angeles is home to about eight percent of the foreign-born population nationwide and 34% of the foreign-born community in California(Ruggles et al., 2020). In Orange County, nearly one-third of adults in the county are foreign born and Orange County’s foreign-born population accounts for 7.2% of California’s total foreign-born population(Ruggles et al., 2020). Informed by input from the RIGHTS Study Community Advisory Board (CAB) and Technical Advisory Committee (TAC), we focused the qualitative portion of the study on specific subgroups so as to avoid treating the diverse Latinx and Asian immigrant communities as homogenous. This included Mexican, Chinese, and Taiwanese immigrants. We chose these communities as they comprise the largest immigrant nationalities from Latin American and Asia; nationally, Mexico (25%) and China (6%) represent the top two birthplaces for U.S. immigrants(Budiman, 2020, p. 2020), and these groups also represent California’s largest Latinx and Asian subgroups ((Ruggles et al., 2020).

The TAC comprised 10 academic researchers focused on immigrant populations and the CAB comprised 7 community-based organizations serving immigrants in both LA and OC counties and at the state level. The members of the committees are listed on the RIGHTS study website.(“The RIGHTS Study” 2018) We consulted with members of both committees to inform our study design, research methods, recruitment and engagement with immigrant participants in research, as well as to receive feedback regarding results and interpretation of the RIGHTS study data. The members of the research team had expertise in areas of health policy or immigrant health, including lived experience as Chinese and Mexican immigrants. The project manager, a Latinx researcher, provided uniform training to Latinx and Chinese research on qualitative methodology and interview skills. The make-up of the team, including researchers’ positionality, is also detailed on the RIGHTS study website and earlier publications(“The RIGHTS Study” 2018; Chen et al., 2021).

## 3. Data collection

The research team both received referrals, and directly recruited, from diverse local community organizations, including the Mexican consulate, ethnic community centers, student resource student centers for undocumented/DACA students, churches, and county health fairs. Recruitment efforts were supplemented by snowball sampling. Participants were eligible to participate if they met the following criteria: 1) Chinese, Taiwanese, or Mexican immigrant ≥18 years old; 2) had a healthcare need; 3) resided in Los Angeles or Orange Counties; and 4) spoke Spanish, Mandarin, Cantonese, or English. In-person interviews were conducted between August 2018 and May 2019. All participants provided verbal consent to participate. Participants received a $25 gift card for their participation.

Interviews lasted approximately 1 h. A semi-structured interview guide was developed through discussion and consensus of our research team and input from our CAB and TAC groups. The interview guide asked about reasons for migrating to the United States, employment experiences, knowledge about immigrants’ rights, and barriers to health care. Following each interview, the interviewer completed a demographic questionnaire to document information about the participant’s place of birth, age, gender, and years in the US, and then recorded observational field notes about their initial impressions and insights. After the interviews were transcribed and translated, team members reviewed these data and drafted more analytical and interpretive memos.

### 3.1. Data analysis

**Fig. 1.**
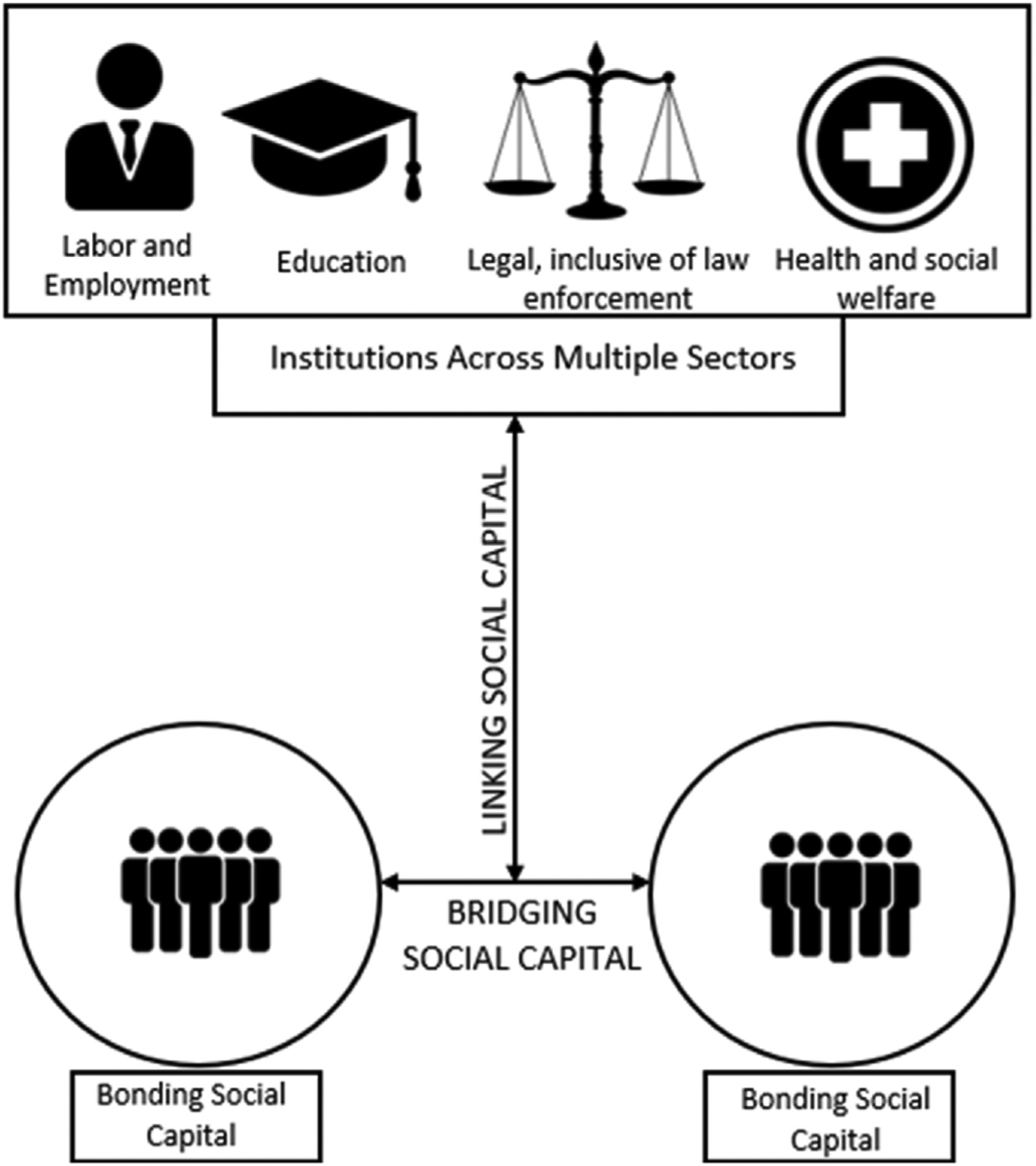
Visualization of conceptual framework.

A constructivist grounded theory approach was used to systematically analyze our data(Strauss & Corbin, 1998). First, interview data were coded using pre-defined codes for each sector: employment, education, law enforcement, and health and social services. Then, the data were examined to compare similarities, differences, and recurring concepts and/or themes within each sector. Social capital theory was used to guide our understanding of how different policy institutions may influence immigrant outcomes. We examined three types of social capital: linking (vertical links to institutions across sectors) and bridging and bonding social capital (horizontal links between friends, family and neighbors and between different demographic and spatial groups, respectively)(Szreter & Woolcock, 2004) (Fig. 1).

To promote reliability, two members of the research team coded the transcripts independently and a third reconciled the codes to generate a codebook. Feedback about coding and consensus about disagreements were reached among the entire research team at weekly team meetings. All coders completed post-coding memos and memos were similarly coded using the codebook generated. All participant names were replaced with pseudonyms to protect their identity. We used Dedoose data analysis software, version 8.3. All research procedures were approved by the Institutional Review Board of [institution redacted for peer review].

## 4. Results

The sample comprised 60 participants (n = 32 Mexican, n = 21 Chinese, n = 7 Taiwanese). Of the 28 Chinese and Taiwanese participants, 15 lived in LAC and 13 lived in OC. Of the 32 Mexican participants, 17 lived in LAC and 15 lived in OC. The mean age of participants was 42 years-old, majority were female (n = 45), and most had >10-year tenure in the US (Table 1). Thirty interviews were conducted in Spanish, 21 in Mandarin, 7 in English, and 2 in Cantonese. Notably, the Chinese and Taiwanese participants were mostly authorized permanent residents while the Mexican participants were mostly undocumented.

Immigrants in our sample described lack of linking social capital across sectors, with a range of subsequent negative consequences on bonding, and bridging social capital. These negative consequences included the significant time and effort required to navigate various sectors and other consequences like loss of needed healthcare services, avoidance of law enforcement despite perceived need for protection and safety (e.g., domestic violence), and loss of employment or educational opportunities. Cultural differences between groups did not emerge as salient in how participants accessed or engaged with different types of social capital. Within this larger narrative of a perceived lack of social capital ties across sectors, we identified four themes pertaining to the gaps in, and uses of, social capital among the immigrants in our sample: (1) Perceived lack of linking social capital in the healthcare sector relative to other sectors; (2) Perceived stigma, fear, or pride in self-sufficiency limiting use of linking social capital; (3) Language was a main barrier to linking capital, with bonding and bridging social capital used to overcome that barrier; and (4) Bonding social capital as a strain and challenge for some immigrants, rather than facilitative or helpful, resulting in reliance on bridging and linking social capital. We discuss these themes with illustrative quotes below (summarized in Table 2).

### (1) Perceived lack of linking social capital in healthcare sector relative to other sectors

Participants gave multiple examples of linking social capital across legal, education, employment, and health sectors – that is, examples of when connections with institutions in one sector facilitated resources to another sector. The education and employment sectors were described as most facilitative of cross-sectoral connections (e.g., counselor at school facilitating access to mental health services or employment insurance facilitating access to health services) and the legal and health sectors as least facilitative. Participants, particularly Mexican immigrants, discussed accessing legal services through community-based organizations (CBOs) that provided support with various legal issues ranging from domestic violence to immigration-related legal counseling. Participants reported experiencing the weakest institutional supports with the health sector.

The following excerpt from one Mexican immigrant participant describes the support she received from a CBO when she was leaving a domestic violence situation, including connection to a therapist as well as legal assistance. When asked if she had to pay for those services, she said “*No, they offered everything without having any medical insurance and* … *they* supported *me a lot*.” CBO support was particularly salient when it came to immigration-related paperwork, e.g., Deferred Action for Childhood Arrivals (DACA) applications or naturalization paperwork. One Mexican DACA-recipient described how she sought out a connection to a CBO to “*help me fill out the paperwork. Why? Because I was afraid of doing it wrong and getting denies. So that’s when I went to [CBO] and they helped me fill out all the paperwork*”

The crucial role of CBOs included faith-based organizations, with one Chinese participant even attending church to access social capital even when not religious.

Interviewer “Do you identify with any religion?”

Participant “No.”

Interviewer “But you go to church because …”

Participant “Because we know people in church will help others out. Also, they speak Chinese. They’re willing to help as much as they can. They told us to come and see what we need. I mean, I asked them to help fill out the enrollment application for my daughter. You know, sometimes you’re forced to come up with the solutions. You need to figure the solutions if there’s no other options.”

Another Chinese participant described her decision to go to CBOs to connect to governmental resources i.e., facilitating access to vertical linking social capital, which was needed due to her marginalized position as an immigrant. In her words:

“I need to rely on myself. But, I didn’t receive much education. I don’t know English, what can I do? Therefore, I chose to go those organizations that aim to provide services to the Chinese community. They’re a bridge between the community and the American government. I was actually very grateful to receive from this place.”

Several participants, both Mexican and Chinese, highlighted how social capital in the employment sector facilitated access to resources in other sectors. For example, one Chinese immigrant described how her employer “

“The owner [from my job] then provided financial assistance for [my daughter] to attend classes,” illustrating connections made between the employment and education sectors. In the case of one Mexican immigrant, the education sector served to facilitate access to resources in the legal sector, including law enforcement, which led to additional benefits by obtaining authorized immigration status.

“I was able to apply [for the U-Visa] through school. My mom told the counselors my story and the school reported it to the police, and they told us we could apply for the U- Visa … And they gave us information. We went to the consulate and [the application] was $1,000 but there was also a grant and we applied, and they approved us so we didn’t have to pay. Since my mom and I are from Mexico, she applied with me, and we had to go to the doctor so that they could make sure I was mentally stable, they needed proof of vaccines … and just this year I received my work permit and social, and that is what has opened many doors … to work and also do my taxes with my husband.”

In the healthcare space itself, therapists, social workers, and resource specialist staff were especially helpful conduits to greater social capital. One Mexican participant described how her mental health provider connected her to a caseworker who then connected her son to mental health services and helped them apply to Medi-Cal (California’s Medicaid healthcare program). In her words:

“So they [mental health provider] gave me the help and assigned me a social worker so she could orient me. She gave me such a good orientation and she recommended some people for the [health] insurance … Sometimes there are angels that are not necessarily your family. They helped me with the health insurance, we got Medi-Cal, and now he [my son] is in the regional center so he gets yearly evaluations.”

Different income levels facilitated access to different benefits and resources, or precluded people accessing those resources. In the case of one Taiwanese participant, for example, she described how her parents “*they pay their tax [in the U.S.] but they are not rich enough. They’re just above the poverty line. They do not qualify for any [public] health insurance. There are a lot of pressures on their adult children and themselves*.” In this case, the absence of linking social capital, hindered by income level, resulted in increased reliance on bonding social capital.

Bridging and bonding social capital were critical to navigating all sectors, but especially healthcare given the weakest institutional supports within this sector. For example, participants described individuals outside the healthcare space itself facilitating access to the healthcare sector i.e., from *promotoras* or community health workers, or at senior centers and places of worship. Within the healthcare space, participants identified therapists, social workers, and resource staff as particular individuals who facilitated access to resources. Participants also described relying on family and community members s to help with various tasks ranging from translation to negotiating bills, as well as facilitating transferring of healthcare-related knowledge or promotion of healthy behaviors. In the absence of linking social capital, bonding social capital became increasingly relied upon. For example, one Chinese participant described how community members allowed her to find a primary care physician and educated others about preventive annual visits:

“Because of the aunts [older adult women] in our church, they have many concepts of finding a family doctor because they have been in the United States for a long time. They all know that they have to take a medical examination once a year.”

*One Chinese participant said,* “*It is really through friends that I find this [the HPV vaccine] is very important*”*). Another Chinese participant described a cousin helping him navigate exorbitant out-of-pocket medical costs when he could not access health insurance: My cousin helped translate, helped me to negotiate and I gave $9,000, I almost owed [the hospital] $100,000*.” *Finally,* they described the expansion of their networks via social media platforms (i.e., ‘WeChat’ by Chinese participants).

### (2) Stigma, fear, and pride in self-sufficiency limited use of linking social capital

Both Mexican and Chinese immigrants described various barriers to using linking social capital. This included societal stigma about immigrants being perceived as “beggars” or draining government resources influencing sentiment about “*not wanting to use government* support,” alongside fear of negative consequences like public charge considerations affecting future immigration status adjustments, and personally held beliefs and pride about self-reliance and self-sufficiency. These sentiments were predominantly shared more by participants in OC rather than LAC, suggesting influence by local context and “immigrant-friendly” policy climates.

This except from a DACA student describes the societal stigma that he felt immigrants must contend with when considering leveraging social capital to access resources:

“My personal struggles would just be I would say the lack of government support. We’re kind of thrown into this society with very little support, if any. And any help we’re trying to get, it’s seen as a handout or us being beggars and not really trying to work for what we want. It’s like a double-edged sword. Like you’re struggling to get ahead, but without any help. You’re struggling even more, so you’re still seen in this negative picture.”

Fears about public charge were particularly salient among the Mexican immigrants in our sample (relative to Chinese participants who were mostly naturalized or permanent residents) as this Mexican participant described: “*I was scared* … *Sometimes I asked for the WIC coupons. I don’t know if that ever affected me, I don’t know* … *but other programs I didn’t get. I was scared*.”

Another Mexican participant described the complexity surrounding the calculus of seeking help that may hurt oneself or one’s family in the long run:

“It’s very difficult to navigate the system in this country, even when you know where to look for help because in general there’s programs that can help you, but … we’re scared because if you ask for help it will affect you if you want to fix your legal status.”

But this fear of unintended negative consequences was expressed among Chinese participants as well, albeit less frequently, as in this Chinese participant’s experience where her family member discouraged her from applying for Medi-Cal because of potential impact on future job prospects:

“You do not need a white card (Medi-Cal card) because, for example, for your later job. It is not good for you to apply for a job or government benefits. If you are a US citizen, I think there will be no problem to apply.”

Fear of negative consequences when using linking social capital was also described in the legal law enforcement context, undermining trust in these sectors. For example, one Mexican immigrant described a challenging experience with law enforcement who did not protect her or her daughter when she requested support against an abusive father violating a restraining order:

“One day I remember I woke up at 3 am and I went to the window, and I see him [ex-husband] pass by in his car with binoculars with a light watching the house. They [the police] said, ‘well buy a dog, buy a dog so he doesn’t go inside the house.’ Another day … he was standing outside [the house] with a current restraining order. I go and call the police, the police investigated me, investigated my daughter … they did an alcohol test, and test for I don’t know how many things and everything was negative … and the police went outside after a while [to talk to the ex-husband] and came back and said, ‘You know what? I would do the same thing that man is doing, just to see my kids.’ I felt like they threw a bucket of water on me”.

A fear of negative consequences leading to decreased engagement with sectors was echoed by other participants. One Mexican participant said “*Here sometimes you suppress yourself by not going out much, not doing things* … *because they may deport you*.”

Beliefs about self-reliance and self-sufficiency was the limiting factor for other immigrants. This excerpt from a Mexican participant relays the sentiment about participants not wanting to access governmental support due to such beliefs. She describes how her husband refused to have them apply for disability benefits or Medi-Cal when she was pregnant and when her son needed mental health services; her husband stated: “*That’s why I work, I’m not going to let the government maintain us*.’ *I had to ask people to come talk to him* … *they came to talk to him, and they couldn’t change his mind*.”

### (3) Language was a main barrier to linking capital, with bonding and bridging social capital used to overcome that barrier

Participants discussed language as critical to facilitating or hindering their ability to access programs and services across sectors. But while language was a barrier to using linking social capital, bonding and bridging capital was used to overcome this barrier i.e., reliance on co-workers, friends, or family to overcome language barriers.

This excerpt from one Mexican immigrant highlights how interpreter and bilingual provider availability in healthcare settings facilitated her understanding of her son’s health concerns.

“For me it was very important to find a place that [people in the clinic] spoke both languages and communicated directly in Spanish and speaks English [for my son]. So that has really helped me a lot. When I found this clinic, they give so much information and I don’t need to know English.”

Bridging and bonding social capital facilitated access across sectors by overcoming language barriers. In the education sector, participants described relying on their family, friends, and community members to draft personal statements, resumés, or find resources on campus. In the employment sector, participants discussed receiving support from co-workers to communicate with their bosses in English, including to advocate for better hours or work conditions. One undocumented Mexican women described how her coworkers would help her communicate with her boss:

“I would ask them [co-workers] can you help me write this note?” They would tell, you know what, go to Google and search for the translator and send it, and I said, “Okay.” That’s how we started and then he [my boss] told me it’s fine. They always classify us as Mexicans or any other country, that we don’t know. But the truth is I think they are wrong. Although we don’t speak English very well, we know how to do things.”.

Social media platform groups allowed for expansion of bridging and bonding capital to facilitate language access. For example, one Chinese immigrant participant used a WeChat support group to communicate with other Chinese parents with children with autism spectrum disorder. Another participant, a Mexican immigrant, developed a strategy to maintain her employment by asking her daughters to teach her to use translating apps on her phone: “*I told him [boss] you know what? I’m going to send you text messages instead and we will communicate like this. I don’t know how to speak it, but I can read it. So, I would* … *go on Google and look for translation*.”

### (4) Bonding social capital as a strain and challenge for some immigrants, rather than facilitative or helpful, resulting in reliance on bridging and linking social capital

Many participants reported lacking support from family members back home because of the migration experience. However, when bonding social capital was present, it was not always facilitative or helpful. In fact, sometimes bonding social capital drained financial or other resources from the individual and contributed to increased psychological distress.

One undocumented Mexican woman described how the distance between immigrants and their families abroad included both physical and psychological distance: “*They [family] cheer you on from a distance, that I should do my best, and it doesn’t go beyond that. But there’s not much to that. There’s a reality that we live that our family members back in our countries are not aware of*.”

Examples of how strained bonding social capital could be harmful included the pressure and burden to send financial assistance, arranging for immigration sponsorship, and caregiving. Most Mexican immigrants in our sample mentioned financial burdens, whereas Chinese immigrants in our sample discussed burdens more associated with immigration sponsorship and caregiving pressures. The financial burden was sometimes born out of a sense of duty to family and other times out of necessity such as in mixed-immigration-status families where one family member may have access to increased employment opportunities due to their immigration status. This excerpt from one Mexican undocumented immigrant describes this tension, that led him to interrupt his college education in order to provide financially for his family and allow for his younger siblings to attend middle and high school:

“Being home and being in an illegal environment – where your parents are illegal, your siblings are illegal, you are forced to help out financially. You are kind of forced to carry some of that burden … I actually started working mid-semester, so I had to stop going to school. I started working nighttime, and then I started increasing the workload, and then I would go to school in the morning, but then I just kind of burn out. I just had to stop going, just to keep supporting my family, helping them go through whatever they have to go through. Helping my younger brothers go to school, because they were still going to high school and middle school, so I still wanted to help them out and be there.”

Some immigrants stated that this financial responsibility to their family members came out of a sense of familial duty or roles (“*because I was the eldest in the family*”), rather than necessity. In the words of one Mexican immigrant: “*I also send money to my parents* … *not because they are asking me to but because I see they have limitations*.”

Expectations of immigration sponsorship also placed a financial and time burden on bonding social capital, among immigrants who were permanent residents or naturalized citizens in the U.S. One Taiwanese participant, who is a permanent resident (“green card holder”) described how he and his wife traveled between Taiwan and U.S. every half year “*because my daughter is relying on my legal status to get her green card*.” He described this as challenge as he was getting older and without health insurance in the U.S. and may have opted not to return otherwise, whereas maintaining his permanent residency required him to live the majority of each year in the US to maintain his status and thereby, sponsor his daughter.

Some immigrants mentioned their dependents’ (i.e., children and older parents) lack of health insurance in the U.S., causing another layer of financial stress for the entire family. One Chinese immigrant woman described her experience with her uninsured older parents this way:

“It is very troublesome for parents’ coming over … Because my parents do not have health insurance when they come. My mother had acute conjunctivitis a while ago, and we had no insurance at that time. We can only take her to see a doctor at that kind of private clinic. It is actually quite expensive if there is no insurance.”

Many Chinese immigrants reported caregiving pressures. One Chinese immigrant woman described how “*Because both my husband and I are only-childs in our families, it means that we two people have to take care of the four old people, so the pressure is quite big*.” Some Chinese immigrants expressed the sense of powerlessness and guilt that came with caregiving across borders. In the words of one Chinese immigrant participant: “*I could do nothing, except sending them [my parents] back some money. I actually felt very guilty*.”

Bonding social capital was strained within couple dyads, in addition to inter-generationally, limiting how helpful bonding social capital could be. For example, one Chinese immigrant woman described tension with her husband because she had given up a career she loved as a teacher in mainland China: “*The only jobs I can do here are very low-skilled* … *[but] my husband wants to stay here, and my child needs a father, right?*”

Consequent to these strained relationships within the bonding social capital domain, participants describing relying more on other connections within both bonding and bridging social capital domains i.e., friends, classmates, language-concordant landlords, social media platform group members, members of a Church, and even co-ethnic group members who someone would meet on the street. One Chinese immigrant woman described how her classmates, who were not necessarily from her same demographic background, helped her: “*The main help comes from classmates* … *[They] introduced me to a job after graduation*.” *Another Chinese immigrant woman described gaining access to the employment sector through her Church community:* “*This job I have now was through someone at that Chinese church. So, it’s all jobs that I just got from getting to know people*.”

## 5. Discussion

Our study extends our knowledge of how linking, bridging, and bonding social capital are derived across sectors among subgroups of the Asian and Latinx immigrant populations– Chinese, Taiwanese and Mexican immigrants. We find that they experience weakest linking social capital in the healthcare sector, thereby increasing reliance on bridging and bonding social capital to facilitate access and navigation of care. Other sectors, particularly the education and employment sectors, facilitated links to other sectors whereas fewer cross-sectoral connections were made in the legal and healthcare sectors. Bridging and bonding social capital were also deemed critical to overcoming language barriers that limit access to linking capital. However, bonding ties sometimes incurred additional burdens and pressure for the immigrants in our study, rather than being uniformly beneficial. Individual factors like stigma, beliefs about self-sufficiency, and fear relating to hindering future access to immigration status changes (e.g., public charge) and/or other opportunities (e.g., employment) exacerbate the relationship with linking social capital within and across sector.

Notably, we did not find cultural differences as being particularly salient, including between Chinese and Taiwanese participants. Although some existing research has viewed some social capital concepts as culturally specific, other research has suggested that these cultural gaps are narrower. For example, the concept of filial piety—or adult children’s attitudes and obligations toward their parents—has been traditionally associated with Asian cultures and can influence bonding social capital(Pan et al., 2022). But this concept has been reported across cultures(Pan et al., 2022), including *familismo* in Hispanic culture that similarly emphasizes close, frequent interactions, loyalty, reciprocity and provision of support among immediate and extended family members(Almeida et al., 2009). Other studies have found that social capital increases with acculturation (Valencia-Garcia et al., 2012), and that ethnicity as a traditional measure of acculturation plays less of a role in the heterogeneity of immigrants’ experiences than environmental factors such as discrimination and neighborhood characteristics (Roth et al., 2019). In the presence of complex processes that interact to influence health, our qualitative study highlights how social structures like local policies and institutions can influence patterns of social capital engagement across different ethnic groups.

Our paper offers a unique cross-sectoral approach, which is critical because state and local legislation extend rights to, or exclude, immigrants through a variety of policies that are not limited to one sector alone i.e., affecting their health care access, education, workplace and more (Wallace et al., 2018; Wallace & de TrinidadYoung, 2018). But while earlier studies have created composite scores to determine the degree of “pro” or “anti-“ immigration-related policy(De Trinidad Young and Wallace 2018), there has been no in-depth qualitative work to assess how this is experienced, including differences across sectors that may inform cross-sectoral interventions. For example, our study suggests that the healthcare sector could benefit from cross-sectoral engagement and referral. Engagement with any one sector could represent a first point of encounter for many immigrants who face a multiplicity of barriers, and thereby play a pivotal role in facilitating institutional engagement (Saadi et al., 2019). Further, our study suggests that it is not enough for healthcare to become a one-stop-shop for all social needs because immigrants may not come into contact with the healthcare sector immediately. Rather, our findings call for bolstering public sectors independently and improving cross-sector collaboration. Future studies should continue to explore the best ways to facilitate cross-sectoral collaboration, referral pathways, and enhanced services offered to patients to address social needs identified in the healthcare space.

Further, our findings underscored how bonding and bridging social capital are increasingly relied upon when linking social capital is absent, particularly in the context of language barriers. Institutions across sectors can therefore bolster interpreter services as well as improve workforce diversity to ensure provision of language-concordant services and enhance leveraging of linking social capital for immigrant populations. Policy level solutions informed by linking social capital could include adjustments to reimbursement for interpreter services (Ku & Flores, 2005).

Indeed, even with linking social capital present, our study highlights personal and policy level factors that need to be addressed to facilitate access. Policy level factors that contribute to fear include public charge that has hindered access to healthcare and social services (Touw et al., 2021), speaking to how policy sectors (i.e., legal and health sectors) are inter-related in the lives of immigrants. Personal factors include perceptions of self-reliance and self-sufficiency, a sense among immigrants that they got this far on their own (with the help of bonding social capital) and they can or should be able to make it with their own effort rather than relying on linking social capital. This may be interrelated with concepts of *machismo* that has been described in Mexican culture (Nuñez et al., 2016), and a common public health “frame” or rhetorical strategy used to endorse immigrants’ inclusion in the U.S. health safety net, i.e., immigrants described as “effortful immigrants” who contribute to society but do not use health services relative to their non-immigrant counterparts (Viladrich, 2012). Furthermore, we saw that the self-sufficiency narrative was more robust among our Orange County subsample, who experience more restrictive policies and climate, rather than Los Angeles County. This suggests that the self-sufficiency narrative, also tied to deservingness or “good immigrant” narratives (Chauvin & Garcés-Mascareñas, 2014), is likely influenced by the social milieu and political context within which immigrants live. Surprisingly, we did not find other differences according to county. It is possible that state policies may mask county policies’ influence when it comes to immigrant social capital, particularly in California where state policies like the California Values Act, limiting involvement of police and sheriffs in federal deportations, had been passed and implemented at the time of this study. In recent decades, state and local law enforcement officers have become the primary frontline agents in U.S. immigration enforcement so such a state-level policy may play a significant role in shaping the local immigration policy climate (Chacón, 2019).

Our study also contributes to existing literature on the potentially harmful aspects of social capital. In particular, our study found family networks resulted in strain, financial stress, and lack of emotional support among some participants. This is in line with transnational research which suggests that family separation may produce financial strain for immigrant women in particular (Afulani et al., 2016). Additionally, there may be discrimination and stigma within immigrant communities. One study, for example, highlighted the stigmatizing identity of undocumented status in the Asian community, leading to workplace exploitation, intra-ethnic conflict, and social isolation among undocumented young adults(Sudhinaraset et al., 2017). While we found evidence that bonding and bridging social capital buffered the lack of linking social capital, particularly in the context of healthcare sector, we found less evidence for linking social capital providing support in the context of family strain and low bonding capital.

Notably, social media platforms emerged as expanding bonding and bridging social capital for immigrants as well as facilitating use of linking social capital across sectors. One study found that participants from a racially marginalized group or who lacked college training were more likely to broaden social networks online rather than face-to-face (Gonzales, 2017). Further, other studies have highlighted that social forums such as these can also be a source of inaccurate, unverified data and information (Naeem et al., 2021). The internet, social media platforms, and social networking sites are re-defining our definitions of “community” and producing new concepts in social capital that includes online social capital, or the ties that are derived from online engagement.(Spottswood, Erin, & Donghee Yvette Wohn, 2020) Future research can explore best methods to leverage these sources to reach immigrants and service their needs, as well as address any associated misinformation. Additionally, future studies should examine the role of online social capital among immigrant communities, for which the internet may play a particular critical role for those less connected to physical institutions.

Our study findings should be understood within its limitations. First, we were unable to ideally identify comparisons between groups due to differences in immigration status i.e., the Mexican immigrants more likely to be undocumented than the Chinese and Taiwanese immigrants. Because comparisons may have been a consequence of immigration status rather than cultural differences, differences based on country of origin, we chose to focus on similarities in experience across immigration status strata. Future studies should explore within- and between-immigrant community differences in the use of individual and institutional/structural social networks. Second, this study was not designed at the outset to explore social capital, so we are limited by what emerged in the interviews. Future work can explore differences in social capital in more detail and further identify mechanisms by which social capital can be protective to individual and community-level health. Despite these limitations, this study contributes to the body of knowledge on the role of social networks among Mexican, Chinese, and Taiwanese immigrant communities and across sectors. To our knowledge, this is the first qualitative study to include distinct immigrant communities and to explore themes across sectors.

In conclusion, we need to view social capital as a modifiable social resource that can be used in promoting health and well-being. To address immigrants’ needs comprehensively, there is need for cross-sectoral engagement as well as advocacy for policy changes that facilitate institutional supports and the building of social capital for immigrant communities including linking, bridging, and bonding social capital. While social capital interventions remain scarce (Villalonga-Olives et al., 2018), this study provides evidence for the ways in which multi-level interventions – across institutions, communities, and individuals – may facilitate access to services and information. While there are few intervention studies that have focused on linking social capital, examples include physical exercise programs in workplaces and early childhood intervention programs (Andersen et al., 2015; Shan et al., 2014). Future studies should develop and evaluate intervention programs that can improve linking, bridging, and bonding social capital for immigrants in particular. Public health practitioners can also pay attention to the ways in which facilitation may occur outside traditional service sectors, including online resources. For example, applications and websites like Tarjimly, Arrived, and Notifica provides immigrants with information on immigration raids and safe passages (Sudhinaraset et al., 2021). Ultimately, structural level interventions that facilitate multi-sector engagement is needed to improve immigrant health and communities.

## Figures and Tables

**Table 1:** Sample characteristics of 2018–2019 RIGHTS interviews, by county of residence and national origin (N = 60).

	Total	Los Angeles County		Orange County	
		Mexican	Chinese and Taiwanese^a^	Mexican	Chinese and Taiwanese^a^
Total	N = 60	n = 17	n = 15	n = 15	n = 13
**Age**					
Mean (years)	42	41	43	43	42
**Gender**					
*%* Female	45 (75%)	13 (76.5%)	12 (80%)	10 (66.7%)	10 (76.9%)
**Time in the US**					
Mean (years)	19	22	15	24	16
**Legal Status**					
% Naturalized citizen	18 (30%)	5.8%	33.3%	33.3%	46.1%
% Permanent resident	15 (25%)	11.8%	53.3%	6.66%	30.8%
% Temporary Status^b^	9 (15%)	29.4%	13.3%	6.66%	7.7%
% Undocumented	18 (30%)	52.9%	-	53.3%	7.7%
**Language of Interview**					
% Cantonese/Mandarin	21(35%)	-	73.3%	-	77.9%
% English	9 (15%)	5.9%	26.7%	6.7%	23%
% Spanish	30 (50%)	94%	-	93%	-
**Undocumented family/friends**					
%Yes	38 (63%)	88.2%	26.7%	100%	30.8%

Notes:

a Includes those from Hong Kong and Taiwan.

b Temporary statuses include: DACA, H1B, L1/L2, and U-Visa.

**Table 2:** Main themes and illustrative quotes.

Main Themes	Illustrative Quotes:
1. Perceived lack of linking social capital in healthcare sector relative to other sectors	“The owner [from my job] then provided financial assistance for [my daughter] to attend classes. ” “I was able to apply [for the U-Visa] through school. My mom told the counselors my story and the school reported it to the police, and they told us we could apply for the U- Visa. ” “Because we know people in church will help others out. Also, they speak Chinese, so I call them to ask if they can help us … I asked them to help me fill out the enrollment application for my daughter. ”
2. Stigma, fear, and pride in self-sufficiency limited use of linking social capital	“I’m embarrassed to ask for help. I don’t want them to take my kids away. That was always my fear: that if I asked for help, they are going to take my kids away, they are going to ask me, why do I want papers? No dear God, just leave it like that. ” “I never liked doing that [getting public benefits] because I was scared. Sometimes I asked for the WIC coupons, it was the only thing I asked for, WIC, it would help me with the milk for the kids … They used to say that wouldn’t affect you and well, yes, I did get it, but other programs I didn’t get. I don’t know … I was scared. ”
3. Language was a main barrier to linking capital, with bonding and bridging social capital used to overcome that barrier	“For me it was very important to find a place that [people in the clinic] spoke both languages and communicated directly in Spanish and speak English [for my son]. So that has really helped me a lot. ” “I asked them [school] to help solve the problem, talk to the teacher, see what can be explained, many others have helped read the letters, because they are all in English. We ask the [people from church] for help. Because we can’t communicate. ”
4. Bonding social capital as a strain and challenge for some immigrants, rather than facilitative or helpful, resulting in reliance on bridging and linking social capital	“I ended up going to community college for about a year, but during that time, being home and being in an illegal environment, where your parents are illegal, your siblings are illegal, you are forced to help out financially. So, you’re forced to carry some of that burden. So, in my example, I actually started working mid-semester, so I had to stop going to school. ” “Because both my husband and I are the only child in our families. It means that we two people have to take care of the four older people. So the pressure is quite big. ”
